# University support and online learning engagement during the Covid-19 period: The role of student vitality

**DOI:** 10.1016/j.heliyon.2023.e12832

**Published:** 2023-01-10

**Authors:** Edem M. Azila-Gbettor, Martin K. Abiemo, Stanley Nelvis Glate

**Affiliations:** a Department of Management Sciences, Ho Technical University, Ghana; b Department of Marketing, Ho Technical University, Ghana

**Keywords:** Covid-19, University support, Online learning engagement, Student vitality

## Abstract

The study investigates the moderating role of students' vitality on the nexus between university support and online learning engagement among tertiary students during the era of Covid-19 pandemic. A sample of 310 business students chosen randomly completed a self-reported questionnaire for the research. Data processing and analysis were done using Statistical Package for the Social Sciences (SPSS) version 24 and SmartPLS 3.3.9, respectively. Results reveal that university support positively and significantly predict students' online learning engagement. Furthermore, students' vitality enhances the positive effect of university support on students’ online learning engagement. This study appears to be one of the first to have investigated a model linking university support, online learning engagement and student vitality from the perspective of higher institutions of learning. The findings suggest higher education managers must build students' states of vitality in order to enhance their online learning engagement during periods of pandemic.

## 1 Introduction

The Covid-19 pandemic is one of the world's worst health catastrophes, affecting people across the world [1]. As of May 5, 2022, an estimated 6.3 million people have died globally from 514 million confirmed cases contracted from five known variants, namely Omicron, Delta, Gamma, Beta, and Alpha [2]. Current studies suggest the pandemic has led to a wide range of negative psychological repercussions among people, including fear [3], death distress [4], anxiety, stress, depression [[5], [6], [7]], and exhaustion [8].

The emergence of Covid-19 has radically transformed the landscape and the lives of students in the higher institutions of learning. Universities have gone into lockdown, and these closures have disrupted regular classes [9]. By April 1, 2020, there were 1.598 billion students in 194 nations who had to stay at home because their educational facilities were closed, regardless of grade level [10]. The unpredictability of Covid-19 scourge has forced institutions of higher learning to convert to an online learning mode in the face of inadequate preparation in terms of provision of information communication technology infrastructure, internet connectivity, instructor capacity, and student-parent preparedness [[11], [12], [13], [14], [15], [16]]. The findings of recent Chinese research revealed that 1,454 colleges have implemented online teaching, with more than 1.18 billion university students enrolled in more than 7.1 million online courses [17]. The transition to online learning also had rippling effects on both students and lecturers psychologically [18,19]. Following the popularisation of online teaching, many researchers and practitioners have begun to worry about the effectiveness of students’ engagement online [20], especially in the areas of quality student experiences [21], attainment of desired learning outcomes [22], academic achievement [23], and intensity and participation of students in educational activities [24] in order to avoid alienation [25], disengagement and student dropout [26].

Prior research has found that support received by students during their studies is an empowering factor in enabling them to meet their university challenges [27,28]. In terms of student online learning engagement, varying forms of support have been identified as significant indicators of students' outcomes [20,29,30]. For example, Gao et al. [20] found family support in the form of emotional, capability, and environmental support to positively predict the e-learning engagement of 1,317 college students in China. In another study, Kara [31] dicovered that the absence of peer support negatively affects the online engagement of 44 university students. This study examined university support as a predictor of students’ online engagement, a nexus that remains unexplored in the context of higher education worldwide.

Despite the fact that support in any form is predicted to have a beneficial effect on students' online engagement, it is conceivable to propose that this relationship may be neutralised owing to the devastating effects of the Covid-19 pandemic. Accordingly, an examination of boundary conditions that might strengthen the effect of university support on students' online learning engagement while also mitigating the exogeneous effect of Covid-19 pandemic must be of paramount interest. This study introduces student vitality as a moderating variable to enhance the positive relationship between university support and online learning engagement. Student vitality represents their sense of being alive, vigorous, and energetic, and is a crucial determinant of overall motivation and wellbeing [32]. The study proposes that students' vitality may provide a buffer [33] to enhance the positive effect of university support on students' online learning engagement. Taking the above logic into consideration, the purpose of the study is to investigate the moderating role of students' vitality in the association between university support and students’ online learning engagement. Explicitly, the study aims to explore the following two objectives.

1. Assess the effect of university support on students' online learning engagement.
2. Assess the moderating effect of student vitality on the nexus between university support and online learning engagement.

This study contributes to the online learning engagement literature as follows. First, studies have highlighted the role of university support in fostering students’ effective online engagement. The finding validates earlier studies which found other forms of support received by students as a significant predictor of online learning [20,29,30]. Second, the study further offers evidence to support the moderating role of student vitality in enhancing the positive effects of university support on online learning engagement in a context of severe epidemic, a relationship which remains unexplored.

## 2 Literature review

### 2.1 University support

The range of support services available to students at higher education institutions is extensive and diverse [34]. In general, university support refers to the system of resources that assist stakeholders, such as professors and students, in carrying out their fundamental responsibilities in an extremely effective and productive manner [35]. University support in terms of online learning can be defined as the provision of technological infrastructure and digital resources to facilitate effective online learning. Studies have revealed that the support provided by universities in the form of technological infrastructure and instructional support is essential to the successful implementation of quality online teaching and learning [36,37]. Other scholars highlight the need for university support in the provision of competent faculty [[37], [38], [39], [40], [41]]. Kebritchi et al. [42] contend that universities must provide support through the provision of technical and pedagogical skills for the paradigm shift in the online environment.

### 2.2 Online learning engagement

There is a plethora of literature on engagement in the context of students. Student engagement is described to cover a wide range of activities, however, it is generally considered to be a ‘complex construct’ [43]. According to Ref. [44], student engagement is an intriguing and multidimensional meta-construct that which is context-dependent and does not occur in a vacuum. Meanwhile, Kahu and Nelson [45] promoted a sociocultural conceptualisation of engagement in higher education. Kuh [46], cited in Ref. [47], refers to student engagement as the phenomenon of student participation in effective practices which take place within and outside the classroom.

Abbas [48] defines online learning engagement as “active participation in e-learning activities facilitated by an e-learning platform”. The term “online learning engagement” describes the enthusiastic, contented mindset that a student brings to the online learning experience [49,50]. References [25,47,51] contend that students' success in online learning engagement is contingent upon four fundamental components including effort and skills, connection to the course material, participation and interaction with peers and teachers, skills, and attainment of desired goals. Online learning engagement has been found to enhance learners’ learning opportunities and autonomy over their open learning [52,53] and improve educational equity [54]. Contrarily to the benefits exposed above, Dumford and Miller [55] found online learning engagement to reduce collaborative learning, and student faculty interaction.

### 2.3 University support and online learning engagement***

Several scholars asserted that students' online learning engagement is feasible and increases with the provision of reliable support in the form of appropriate technological platforms and digital resources by higher learning institutions [[56], [57], [58], [59]], flexibility in the learning process, and effective interaction between students and lecturers [56,60,61]. In a critical review of the literature, Henrie et al. [62] argued that students' online engagement will be greatly enhanced when there is deliberate institutional support for e-learning. Even though an empirical study on the direct nexus between university support and online learning engagement is non-existent, other studies have confirmed a positive link between other dimensions of support and online learning engagement. For example, Luan et al. [63] reported a positive link between social support (measured using peer and teacher support) and online learning engagement among 615 university students in China. Therefore, it is feasible to conclude that students’ online engagement may be greatly enhanced when the institution offers them the appropriate support. Based on this evidence, the ensuing hypothesis is proposed:

H1 There is a positively significant link between university support and online engagement among students.

### 2.4 Moderating role of student vitality

Subjective vitality has been defined as the energy that is accessible to the self in order to harness and manage intentional activity [64], and it is seen as a prominent and functionally relevant marker of well-being and drive [65]. Subjective vitality is also conceptualised as the sensation of having positive energy at one's disposal or under one's own regulatory control [66]. It is an overall sense of mental and physical vigour, excitement, and a zest for life [67]. Ryan and Deci [68] posit that subjective vitality is built on three key psychological innate needs, namely, competence, autonomy and relatedness. According to Ref. [64], activities that meet these fundamental psychological demands should help to maintain or improve individuals' subjective vitality. Given that subjective vitality is built on the idea that one's own energy is always accessible to oneself, it is expected to encourage voluntary activity and performance as well as other beneficial consequences [69].

Student vitality is coined after subjective vitality and is described as the physical state of aliveness that supports students' to attain or fulfil their real sense of purpose in education [70]. High levels of psychological needs fulfilment and low levels of needs frustration have been linked to increased vitality among students [71,72] and frustrated by controlling school environment [65]. Additionally, Blackwell et al. [32] reported student vitality to be dependent on differences in the quality and style of teacher-student relationships, while [73] highlighted gratitude and resilience as predictors of students' vitality. Ryan and Deci [74] posit that students feel vital if successfully “in sync” with their inner selves. Miksza et al. [75] found vitality among university students to correlate positively with their adaptability to adversity and the quality of interactions with peers, and negatively with overall stress experiences. In another study [76], reported vitality to positively predicted life satisfaction, life skills and psychological well-being among 360 students at Bandar Abbas Azad University in Iran. Other studies have discovered a strong positive nexus between students’ vitality and self-efficacy [77], subjective happiness [78,79], motivation and self-confidence [76] and positive mental health [80]. Contrarily, a few studies have also found a negative association between student vitality and burnout [77] and psychological distress [66].

Based on the review, the research suggests that the relationship between university support and online learning engagement would be significantly positive, especially when universities provide the required enabling environment for e-learning. Given that the Covid-19 scourge was a significant hindrance to students’ activities, the study explores student vitality as a boundary condition to enhance the positive link between university support and online learning engagement. The study contends that students high on vitality are more likely to be motivated to deal with their adversity during the Covid-19, and become focused and enthusiastic about participating in meaningful educational activities [81].

The moderating role of student vitality can be explained using self-determination theory (SDT). According to the SDT, three essential and universal psychological needs, such as competence, autonomy, and relatedness, drive people to grow and evolve [68]. SDT differentiates between controlled and autonomous regulation, with the former perceived to be externally regulated and the latter seen as volitional and self-endorsed [82,83]. According to the SDT, regulated forms of motivation deplete available energy and reduce subjective vitality, while autonomous activities may preserve or even increase available energy [69]. Student vitality represents an autonomous regulation that fulfils the psychological need for internal motivation with exciting and empowering energy that may allow students to persist in important educational activities including online engagement during the Covid-19 pandemic [1]. The study suggests that in the period of Covid-19, students’ vitality might play a critical role in the regulation of their purposeful actions, which may in turn enhance their online learning engagement. Based on the above review, it is hypothesised that:

H2 Student vitality would positively moderate the direct nexus between university support and online learning engagement.

**Fig. 1:**
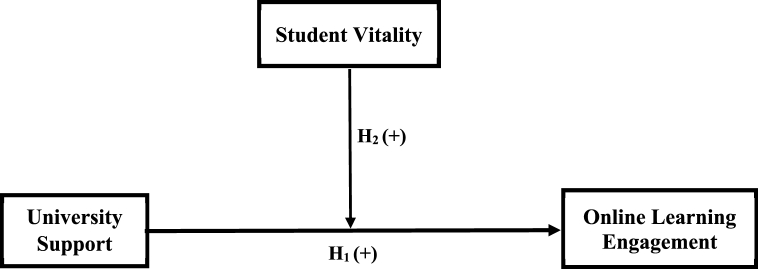
Moderating model of University Support, Online Learning Engagement and Student Vitality.

The model is an illustration of the anticipated links between the three variables (Fig. 1).

## 3 Methods

### 3.1 Participants and procedure

A cross-sectional design was used to test the study model. The population of the study consists of 1900 full-time students pursuing business programmes at a Technical University in Ghana. A sample size of 320 was used [84]. A sample frame created from the institution's registry was used to select the respondents using a simple random sampling procedure. A simple random sampling technique was used to offer every student an equal chance and to reduce high refusal rates [85,86]. Three hundred and fifteen self-reported questionnaires were returned and 310 were found to be useful after editing, resulting in a 96.9% response rate. Data collection was supervised by four trained research assistants and took place between November and December 2020. Prior to distributing surveys, respondents' approval was sought. In addition, participants were informed that the information they provided would be kept private and confidential. The university's Publication and Ethics Review Committee gave their stamp of approval to the study, with protocol number PERC 034, and it followed all the guidelines set out in the Declaration of Helsinki for research involving human participants.

### 3.2 Measures

There were two main sections of the questionnaire. Data on respondents' marital status, age, gender, birth year, and programme were collected in Section A. Section B gathered information on the variables used in the study including university support, student vitality and online learning engagement. Measures on university support were adapted, while those on student vitality and online learning engagement were adopted. The variables were measured using 37 items (Table 1 [[25], [66], [87]]). Fifty students from a nursing training college served as a pilot group for the survey. The reported Cronbach alpha values from the pilot test are university support (0.742), student vitality (0.811), and online learning engagement (0.832). A 5-point Likert scale with a range of 1 (*strongly disagree*) to 5 (*strongly agree*) was used to score each item. Sample items of measures include **(i) *university*** support **-**“*If students are having difficulties with their academic coursework, they can easily talk to lecturers or their teaching assistants”, (ii)* ***student vitality***- “*I feel energized*” and (ii) ***online learning engagement* -** “*I am able to participate actively in small-group discussion forums”.*

**Table 1:** Sources of measures of concepts.

Latent Construct	Source	No. of Items	Range of Scale
*University support*	[87]	*11*	5-point LS; **1** (S*trongly disagree) to***5** (S*trongly agree)*.
*Online learning engagement*	[25]	19	5-point LS; **1** (S*trongly disagree) to***5** (S*trongly agree)*.
*Student vitality*	[66]	7	5-point LS; **1** (S*trongly disagree) to***5** (S*trongly agree)*.

### 3.3 Analytical approach

For the processing of the data, IBM SPSS statistical version 24.0 was used. The respondent's profile was analysed using the frequency and percentages. The study model and related hypotheses were verified using SmartPLS 3.3.9, a Partial Least Square-Based Structural Equation Modelling (PLS-SEM) software. The values of the model fit, discriminant validity, adjusted R^2^, and inner Variance Inflation Factor (VIF) measurement models were determined using the PLS algorithm. To find the direct route coefficients as well as their significant levels, the structural model was run through 10,000 re-samples. The moderating analysis was performed using PLS-SEM product indicator approach [88].

## 4 Results

### 4.1 Profile of respondents

As shown in Table 2, 51.9% are females and 42.1% are males. Exactly 50.3% of the respondents are aged between 21 and 25 years and 86.1% are single, 39.7% are in the third year of their studies (Table 1). The distribution of respondents with regard to age and marital status is representative of the profile of university students in Ghana [38,[89], [90], [91], [92], [93]].

**Table 2:** Demographic profile of sample.

Characteristic		Frequency	Percent
Gender	Male	149	48.1
	Female	161	51.9
Age	≤20 yrs.	31	10.0
	21–25 yrs.	156	50.3
	≥26 yrs.	123	39.7
Marital Status	Married	43	13.9
	Single	267	86.1
Year	Level 100	76	24.5
	Level 200	111	35.8
	Level 300	123	39.7

### 4.2 Measurement model assessment

The validity and reliability of the coefficients of latent constructs were used in the assessment of the quality of the measurement model. Based on the findings of the latent constructs, it is presumed that the model is appropriate for structural analysis [Table 3] [94,95]. Explicitly, the Composite Reliability (CR) coefficients varied from 0.887 to 0.903, above the suggested upper limit of 0.70 [96]. Additionally, the Cronbach alpha (CA) values varied from 0.830 to 0.876, above the suggested maximum of 0.7 [97]. Additionally, all variables' Average Variance Extracted (AVE) coefficients were greater than 0.50, ranging from 0.538 to 0.662, proving the model's latent variables' convergent validity and dependability [98].

**Table 3:** Factor loadings, validity and reliability of latent constructs.

Constructs and Items	Loadings	VIF	CR	CA	AVE
University Support (US)			0.897	0.857	0.636
US_1_	0.769	1.813			
US_2_	0.852	2.379			
US_3_	0.820	2.156			
US_4_	0.772	1.804			
US_5_	0.772	1.721			
US_7_	0.779	1.120			
US_8_	0.733	1.214			
US_10_	0.843	1.111			
US_11_	0.738	1.132			
*Online Learning Engagement* (OLE)			0.903	0.876	0.538
OLE_1_	0.769	2.190			
OLE_2_	0.735	1.811			
OLE_3_	0.856	1.658			
OLE_4_	0.882	1.660			
OLE_6_	0.763	2.219			
OLE_8_	0.818	2.466			
OLE_10_	0.758	1.956			
OLE_11_	0.773	1.533			
OLE_13_	0.769	2.190			
OLE_15_	0.735	1.811			
OLE_16_	0.756	1.658			
Student Vitality (SV)			0.887	0.830	0.662
SV_1_	0.782	1.571			
SV_2_	0.828	1.927			
SV_4_	0.849	2.034			
SV_5_	0.795	1.757			
SV_6_	0.782	1.571			

**Notes**: CA, Cronbach's Alpha; CR, Composite Reliability; AVE, Average Variance Extracted; VIF, Variance Inflation Factor.

The discriminant validity was evaluated using Fornell-Larcker and Heterotrait-Monotrait (HTMT) criteria [88,99]. As observed in Table 4, the square root of all constructs' AVEs in the matrix diagonal is larger than the related correlations in the corresponding columns and rows, showing the reflective model's quality [100]. For instance, the AVE for student vitality (0.814) is greater than the corresponding row correlation (0.702) and column correlation (0.445). Accordingly, study model's latent variables are different, thus signifying the quality of the measured construct. Additionally, the HTMT values of all constructs is less that the recommended limit of HTMT_0.90_ [99], showing that the three latent variables that were used in the study were conceptually distinct from one another.

**Table 4:** Discriminant validity.

Fornell-Larcker Criterion	OLE	SV	US
Online Learning Engagement (OLE)	0.734		
Student Vitality (SV)	0.702	0.814	
University Support (US)	0.455	0.445	0.798
*Heterotrait-Monotrait Ratio (HTMT) Criterion*			
Online Learning Engagement (OLE)			
Student Vitality (SV)	0.819		
University Support (US)	0.517	0.526	

### 4.3 Model estimation

The standard root mean square residual (SRMR) value was used to evaluate model fit [101]. The SRMR was 0.065 < 0.08, exhibiting a strong model fit [102] (Table 5). The adjusted R2 criteria was used to measure the model's explanatory power (Table 5) [103]. The adjusted R^2^ value reveals 51.4% of the variability observed in students' online engagement is explained by university support and student vitality. Stone-Q^2^ Geisser's Test was used to evaluate the model's predictive validity [104,105]. The Q^2^ value of online learning engagement (0.267) demonstrates medium predictive relevance [106] (Table 5). Further results indicate the magnitude of the effect of university support (*f*^*2*^ = 0.424) and student vitality (*f*^*2*^ = 0.644) on online learning engagement met the effect threshold of medium effect size [107].

**Table 5:** Summary of R^2^, model fit, Q^2^ and collinearity assessment.

Construct Coefficient of Determination (R^2^)	R^2^	Adjusted R^2^	
Online Learning Engagement	0.518	0.514	
Model Fit	**Value**		
SRMR	0.065		
Q^2^	0.267		
Inner VIF Values	**OLE**	**SV**	**US**
Online Learning Engagement (OLE)			
Student Vitality (SV)	1.247		
University Support (US)	1.247		

The collinearity among the independent constructs was assessed using VIF prior to testing of the hypotheses [108]. Results in Table 5 show that there is no collinearity among predictors of online learning engagement since the VIFs are below 3.

Results of the hypotheses are presented in Table 6. *H*_*1*_ is supported since data confirm a significantly positive link between university support and online learning engagement (*β* = 0.976; *t* = 11.183; *p* = 0.000). The results indicate that when university administrators offer the necessary technical infrastructure for online learning, student participation in online learning is viable. *H*_*2*_ was supported since the moderation of the nexus between university support and online learning engagement by student vitality was positive and significant (*β* = 0.530; *t* = 8.432; *p* = 0.004). This suggests the relationship between university support and online learning engagement is enhanced when they experience high vitality.

**Table 6:** Assessment of direct and indirect hypotheses.

Hypothesis	Path	Path Coefficient	T Statistics	P Values
H_1_	University support - > Online learning engagement	0.976	11.183	0.000
	*Moderating effect of student vitality on;*			
H_2_	University support - > Online learning engagement	0.530	8.432	0.004

p-values significant @ 95%.

## 5 Discussions

The study investigates the nexus among university support, online learning engagement and student vitality amongst 310 tertiary education students. Consistent with earlier studies, university support positively influences students' online learning engagement [37,62,[109], [110], [111]], thereby supporting H_1_. The result validates the positive influence of university support on students' online learning engagement. The results imply that to achieve meaningful engagement of students’ online learning, authorities must provide an efficient online learning environment for students, including the provision of necessary support in the areas of faculty competence, appropriate ICT platform, and adequate digital resources.

The study further examines student vitality as a moderator in the relationship between university support and online learning engagement. Student vitality moderates the nexus between university support and online learning engagement, thereby supporting hypothesis H_2._ The results validate the notion that student vitality has a favourable moderating impact on online learning engagement. Subjective vitality is a sensation of being energized, alive, and free of tiredness and exhaustion [112]. Highly energetic students are more likely to be driven, attentive, and enthusiastic about participating in educational activities [81,113]. The findings from this research confirm student vitality functions as an additional motivational force in increasing students’ level of online engagement, a relationship which has not previously been examined.

## 6 Theoretical and empirical implications

In the higher education literature, the study contributes to existing theory by proposing a moderating mechanism to investigate the influence of university support on students' online learning engagement. The study validates the significance of student vitality in enhancing students' levels of online engagement in the era of pandemic and uncertainty, a relationship that has not been previously examined. The SDT was used to explain the moderating role of student vitality. The application of the SDT deepens the fundamental understanding of how student vitality serves as a further boost for students’ online learning engagement. The findings imply that when students have an inherent energy and motivation to act, the favourable association between university support and online learning engagement is enhanced.

The study highlights the significance of students' vitality in fostering online learning engagement even in the presence of university support. Accordingly, efforts must be directed at assisting students to enhance their state of vitality through the fulfilment of their psychological needs for competence, relatedness and autonomy. For instance, in terms of relatedness, institutions are encouraged to provide environment that supports and enhances relatedness behaviour such as encouraging student collaboration and teamwork [114], fostering an environment of compassion, respect, and cooperation [115], presenting real-time, interactive courses where students may participate or forming small teacher-student support groups [116]. Additionally, techniques to promote the display of respect, gratitude, and empathy during challenging epidemic moments should be offered to students [117]. Furthermore, students should be permitted to collaborate on group projects [118]. Some of the tools to utilise in increasing students' competence include peer or self-evaluation systems, relevant feedback, and communication of clear expectations [119]. Finally, autonomy may be cultivated by allowing students to choose tasks and subjects based on their interests [117]. Additionally, the education system should encourage students to accept responsibility and build a feeling of self-reliance. Vitality-building events may also have a major influence on online learning engagement during pandemics, hence they should be included in the university curriculum. One such intervention is self-reflective practices, which should enable students to keep a journal and write down various activities they have successfully executed even amidst traumatic settings and negative odds. This will help students to appreciate recovery strategies for cognitive and physical workload, thereby fostering students' aliveness, vigour, energy, and overall motivation and wellbeing [32,120], which in turn enhances students’ internal psychological resources to improve their long-term vitality [73].

With regards to university support, authorities must devote time to continual evaluation of online learning quality, course satisfaction, and learner engagement, and utilise the findings of these assessments to inform policy choices. With the new method of delivery, students may be supplied with necessary and relevant online resources and materials to aid their online learning requirements and, as a result, increase their knowledge, abilities, and performance, as well as develop positive attitudes about learning. Universities may also invest in Learning Management Systems and other online learning technologies that are effective, efficient, and simple to use. Further, universities should provide equitable access to student support services regardless of mode, including ongoing orientation that will cover a wide range of the abilities required to manoeuvre through a digital learning environment. Additionally, universities should take appropriate steps to enhance the skills of both teaching and non-teaching through training in instructional delivery approaches and provision of support services to students, respectively [121].

## 7 Limitations and future directions of the study

Firstly, the data used for the study was gathered by means of a cross-sectional approach. The testing of causality between the variables would be hampered by this technique, hence future studies should consider adopting a longitudinal approach. Secondly, this is the first study that examined student vitality as a moderator of the association between university support and students’ online learning engagement. Replication of this study in other educational settings is highly recommended. This will aid in establishing the reliability and validity of the results and enhance the generalization of the results [122]. Similarly, future studies may consider examining student vitality as a moderator between antecedent variables and other student engagement dimensions. The existing model can be extended to either mediation moderation model or moderation mediation model through incorporation of new mediators after vigorous literature search.

## Author contributions statement

**Edem M. Azila-Gbettor**: The study's conception and design, execution, analysis and results interpretation, and writing of the article.

**Martin K. Abiemo:** Designed and carried out the study; instrument design, analytical tools and procedure; and composed the manuscript.

**Stanley Nelvis Glate**: Performed the experiments; materials, analysis tools or data; wrote the paper.

## Funding statement

There was no dedicated grant for this study from any governmental, private, or non-profit funding agency.

## Data availability statement

The data that has been used is confidential and would be provided on request.

## Declaration of competing interest

The authors declare that they have no known competing financial interests or personal relationships that could have appeared to influence the work reported in this paper.

## Additional information

This article has no other information.

## References

[1] Arslan G., Yıldırım M., Aytaç M. (2022). Subjective vitality and loneliness explain how coronavirus anxiety increases rumination among college students. Death Stud..

[2] World Health Organization (2022).

[3] Ahorsu D.K., Lin C.Y., Imani V., Saffari M., Griffiths M.D., Pakpour A.H. (2020). The fear of Covid-19 Scale: development and initial validation. Int. J. Ment. Health Addic..

[4] Yıldırım M., Güler A. (2021). Positivity mechanism explains how covid-19 perceived risk increases death distress and reduces happiness. Pers. Indiv. Differ..

[5] Arslan G., Yıldırım M. (2021). Coronavirus stress, meaningful living, optimism, and depressive symptoms: a study of moderated mediation model. Aust. J. Psychol..

[6] Mensah C., Azila-Gbettor E.M., Amissah E.F., Addison E. (2022). Covid-19, financial anxiety and the psychological well-being of hotel workers. Int. J. Hospit. Tourism Adm..

[7] Taylor S., Landry C.A., Paluszek M.M., Fergus T.A., McKay D., Asmundson G.J. (2020). Development and initial validation of the covid stress scales. J. Anxiety Disord..

[8] Yıldırım M., Solmaz F. (2022). Covid-19 burnout, covid-19 stress and resilience: initial psychometric properties of covid-19 burnout scale. Death Stud..

[9] Schleicher A. (2020). https://www.OECD.org/education/the-impact-of-covid-19-on-education-insights-education-at-a-glance-2020.pdf.

[10] UNESCO (2020). Covid-19 impact on education. https://en.unesco.org/covid19/educationresponse.

[11] Crick J.M., Crick D. (2020). Coopetition and covid-19: collaborative business-to-business marketing strategies in a pandemic crisis. Ind. Market. Manag..

[12] Elshami W., Taha M.H., Abdalla M.E., Abuzaid M., Saravanan C., Al Kawas S. (2022). Factors that affect student engagement in online learning in health professions education. Nurse Educ. Today.

[13] Oraif I., Elyas T. (2021). The impact of covid-19 on learning: investigating EFL learners' engagement in online courses in Saudi Arabia. Educ. Sci..

[14] Salas-Pilco S.Z., Yang Y., Zhang Z. (2022). Student engagement in online learning in Latin American higher education during the covid-19 pandemic: a systematic review. Br. J. Educ. Technol..

[15] Sharma P., Leung T.Y., Kingshott R.P.J., Davcik N.S., Cardinali S. (2020). Managing uncertainty during a global pandemic: an international business perspective. J. Bus. Res..

[16] Spitzer M.W.H., Gutsfeld R., Wirzberger M., Moeller K. (2021). Evaluating students' engagement with an online learning environment during and after covid-19 related school closures: a survival analysis approach. Tren. Neu. Educ..

[17] Huang R.H., Liu D.J., Zhan T. (2020).

[18] Baloran E.T., Hernan J.T., Taoy J.S. (2021). Course satisfaction and student engagement in online learning amid Covid-19 pandemic: a structural equation model. Turk. Online J. Dist. Educ..

[19] Besser A., Flett G.L., Zeigler-Hill V. (2020). Scho. Of Tea. and Lear. in Psych. Adv. onl. Pub..

[20] Gao H., Ou Y., Zhang Z., Ni M., Zhou X., Liao L. (2021). The relationship between family support and e-learning engagement in college students: the mediating role of e-learning normative consciousness and behaviors and self-efficacy. Front. Psych..

[21] Howard S.K., Ma J., Yang J. (2016). Student rules: exploring patterns of students' computer-efficacy and engagement with digital technologies in learning. Comp. Educ..

[22] Manwaring K.C., Larsen R., Graham C.R., Henrie C.R., Halverson L.R. (2017). Investigating student engagement in blended learning settings using experience sampling and structural equation modeling. Internet High Educ..

[23] Cárdenas-Robledo L.A., Peña-Ayala A. (2018). Ubiquitous learning: a systematic review. Telematics Inf..

[24] Azevedo F.S., Sherin B.L. (2012). An evolving framework for describing student engagement in classroom activities. J. Math. Behav..

[25] Dixson M. (2015). Measuring student engagement in the online course: the online student engagement scale. Online Learn..

[26] Owusu-Agyeman Y., Andoh J.S., Lanidune E. (2021). The covid-19 pandemic and student engagement in online learning: the moderating effect of technology self-efficacy. J. Ped. Res..

[27] Martinez-Lopez Z., Tinajero C., Rodriguez M.S., Paramo M.F. (2019).

[28] Rodriguez M.S., Tinajero C., Paramo M.F. (2017). Pre-entry characteristics, perceived social support, adjustment and academic achievement in first-year Spanish university students: a path model. J. Psych..

[29] Klem A.M., Connell J.P. (2004). Linking teacher support to student engagement and achievement. J. Sch. Health.

[30] Wilson D.M., Summers L., Wright J. (2020). Faculty support and student engagement in undergraduate engineering. J. Res. Inn. Tea. Lea..

[31] Kara N. (2021). Enablers and barriers of online learning during the covid-19 pandemic: a case study of an online university course. J. Univ. Teach. Learn. Pract..

[32] Blackwell J., Miksza P., Evans P., McPherson G.E. (2020). Student vitality, teacher engagement, and rapport in studio music instruction. Front. Psych..

[33] Frazier P.A., Tix A.P., Barron K.E. (2004). Testing moderator and mediator effects counseling psychology research. J. Counsel. Psychol..

[34] Farajollahi M., Moenikia M. (2010). The study of relation between students support services and distance students' academic achievement. Proc.-Soc. Beh. Sci..

[35] Al-Enazi G.H.A. (2016).

[36] Kibaru F. (2018). Supporting faculty to face challenges in the design and delivery of quality courses in virtual learning environments. Turk. Online J. Dist. Educ..

[37] Pedro N., Kumar S. (2020). Institutional support for online teaching in quality assurance frameworks. Online Learn..

[38] Azila-Gbettor E.M., Mensah C., Atatsi E.A., Abiemo M.K. (2022). Predicting students’ engagement from hope and mindfulness. J. Appl. Res. High Educ..

[39] Baran E., Correia A. (2015). A professional development framework for online teaching. Tech Tren.: Link. Res. Pract. Imp. Lear..

[40] Beck D., Ferdig R. (2018). Evolving roles of online and face-to-face instructors in a lecture/lab hybrid course. Q. Rev. Dist. Educ..

[41] Garrett R., Legon R., Fredericksen E. (2019).

[42] Kebritchi M., Lipschuetz A., Santiague L. (2017). Issues and challenges for teaching successful online courses in higher education: a literature review. J. Educ. Technol. Syst..

[43] Zepke N. (2018). Student engagement in neo-liberal times: what is missing?. High Educ. Res. Dev..

[44] Appleton J.J., Christenson S.L., Furlong M.J. (2008). Student engagement with school: critical conceptual and methodological issues of the construct. Psychol. Sch..

[45] Kahu E.R., Nelson K. (2018). Student engagement in the educational interface: understanding the mechanisms of student success. High Educ. Res. Dev..

[46] Kuh G. (2003). What we’re learning about student engagement from NSSE: benchmarks for effective educational practices. Chan.

[47] Schreiber B., Yu D. (2016). Exploring student engagement practices at a South African university: student engagement as reliable predictor of academic performance. S. Afr. J. High Educ..

[48] Abbas T.M. (2017). Human factors affecting university hospitality and tourism students' intention to use e-learning: a comparative study between Egypt and the UK. J. Hum. Resour. Hospit. Tourism.

[49] Schaufeli W.B., Salanova M., González-Romá V., Bakker A.B. (2002). The measurement of engagement and burnout: a two sample confirmatory factor analytic approach. J. Happiness Stud..

[50] Wang Y., Cao Y., Gong S., Wang Z., Li N., Ai L. (2022). Interaction and learning engagement in online learning: the mediating roles of online learning self-efficacy and academic emotions. Learn. Indiv Differ.

[51] Czerkawski B., Lyman E. (2016). An instructional design framework for fostering student engagement in online learning environments. Tech Tren: Link. Res. Prac. Imp. Learn..

[52] Carrier M., Carrier M., Damerow R.M., Bailey K.M. (2017). Digital Language Learning and Teaching: Theory, Research and Practice.

[53] Carrier M., Damerow R.M., Bailey K.M. (2017).

[54] Tamah S.M., Triwidayati K.R., Utami T.S.D. (2020). Secondary school language teachers' online learning engagement during the covid-19 pandemic in Indonesia. J. Inf. Technol. Educ..

[55] Dumford A.D., Miller A.L. (2018). Online learning in higher education: exploring advantages and disadvantages for engagement. J. Comput. High Educ..

[56] Abbad M., Morris D., de Nahlik C. (2019). Looking under the bonnet: factors affecting student adoption of e-learning systems in Jordan. Int. Rev. Res. Open Dist. Learn..

[57] Donaldson L., Matthews A., Walsh A., Brugha R., Manda-Taylor L., Mwapasa V., Byrne E. (2019). Collaborative tools to enhance engagement in a blended learning Master's programme. Irel. J. Tea. Lear. High. Educ..

[58] Hew K. (2016). Promoting engagement in online courses: what strategies can we learn from three highly rated MOOCS. Br. J. Educ. Technol..

[59] Mehdinezhad V. (2018). First year students' engagement at the university. Inter. Onl. J. Educ. Sci..

[60] Devine K., Hurst D.C. (2018). On the Line.

[61] Ma J., Han X., Yang J., Cheng J. (2015). Examining the necessary condition for engagement in an online learning environment based on learning analytics approach: the role of the instructor. Internet High Educ..

[62] Henrie C., Halverson L., Graham C. (2015). Measuring student engagement in technology-mediated learning: a review. Comp. Educ..

[63] Luan L., Hong J.C., Cao M., Dong Y., Hou X. (2020). Exploring the role of online EFL learners' perceived social support in their learning engagement: a structural equation model. Interact. Learn. Environ..

[64] Ryan R.M., Deci E.L. (2008). A self-determination theory approach to psychotherapy: the motivational basis for effective change. Can. Psychol..

[65] Ryan R., Deci E. (2017).

[66] Ryan R.M., Frederick C. (1997). On energy, personality, and health: subjective vitality as a dynamic reflection of well‐being. J. Prev..

[67] Arslan G. (2021). Loneliness, college belongingness, subjective vitality, and psychological adjustment during coronavirus pandemic: development of the college belongingness questionnaire. J. Pos. Sch. Psych..

[68] Ryan R.M., Deci E.L. (2000). Intrinsic and extrinsic motivations: classic definitions and new directions. Contemp. Educ. Psychol..

[69] Martela F., DeHaan C.R., Ryan R.M. (2016). Self-regulation and Ego Control.

[70] Stern D. (2016).

[71] Ryan R., Bernstein J., Brown K. (2018). Weekends, work, and well-being: psychological need satisfactions and day of the week effects on mood, vitality, and physical symptoms. J. Clin. Psychopathol..

[72] Ryan R., Rigby C., Przybylski A. (2016). The motivational pull of video games: a self-determination theory approach. Motiv. Emot..

[73] Garg N., Sarkar A. (2020). Vitality among university students: exploring the role of gratitude and resilience. J. Org. Effect.: Peo. Perf..

[74] Ryan R., Deci E., Chirkov V., Ryan R., Sheldon K. (2016). Human Autonomy in Cross-Cultural Context.

[75] Miksza P., Evans P., McPherson G. (2019). Wellness among university- level music students: a study of the predictors of subjective vitality. Mus. Sci..

[76] Fini A.A.S., Kavousian J., Beigy A., Emami M. (2010). Subjective vitality and its anticipating variables on students. Proc.-Soc. Beh. Sci..

[77] Sariçam H. (2015). Subjective happiness and hope. Unisa Psychol..

[78] Uysal R., Satici S.A., Akin A. (2013). Mediating effect of facebook addiction on the relationship between subjective vitality and subjective happiness. Psychol. Rep..

[79] Uysal R., Satici S.A., Satici B., Akin A. (2014). Subjective vitality as mediator and moderator of the relationship between life satisfaction and subjective happiness. Educ. Sci. Theor. Pract..

[80] Salama-Younes M. (2011). Positive mental health, subjective vitality and satisfaction with life for French physical education students. World J. Sport Sci..

[81] Ugur E., Kaya Ç., Özçelik B. (2019). Subjective vitality mediates the relationship between respect toward partner and subjective happiness in teachers. Uni. J. Educ. Res..

[82] Deci E.L., Ryan R.M. (2000). The what and why of goal pursuits: human needs and the self-determination of behaviour. Psych. Inq..

[83] Deci E.L., Ryan R.M. (2008). Self-determination theory: a macrotheory of human motivation, development, and health. Can. Psychol..

[84] Krejcie R.V., Morgan D.W. (1970). Determining sample size for research activities. Educ. Psychol. Meas..

[85] Beins B.C., McCarthy M.A. (2012).

[86] Patten M.L., Newhart M. (2018).

[87] Wintre M.G., Gates S.K., Pancer W.M., Pratt M.S., Polivy J., Birnie-Lefcovitch S., Adams G. (2009). The student perception of university support and structure scale: development and validation. J. Youth Stud..

[88] Fornell C., Larcker D.F. (1981). Evaluating structural equation models with unobservable variables and measurement error. J. Mark. Res..

[89] Azila-Gbettor E.M., Atsu E., Quarshie A.N.K. (2022). Job stress and job involvement among tertiary interns: the buffering role of perceived coworker support. Heliyon.

[90] Azila-Gbettor E.M., Mensah C., Abiemo M.K., Agbodza M. (2022). Optimism and intellectual engagement: a mediating moderating role of academic self-efficacy and academic burnout. J. Appl. Res. High Educ..

[91] Azila-Gbettor E.M., Mensah C., Abiemo M.K., Bokor M. (2021). Predicting student engagement from self-efficacy and autonomous motivation: a cross-sectional study. Cog. Educ. Next.

[92] Azila-Gbettor E.M., Mensah C., Abiemo M.K. (2022). Self-efficacy and academic programme satisfaction: mediating effect of meaningfulness of study. Int. J. Educ. Manag..

[93] Mensah C., Azila-Gbettor E.M., Appietu M.E., Agbodza J.S. (2021). Internship work-related stress: a comparative study between hospitality and marketing students. J. Hospit. Tourism Educ..

[94] Hair J., Hollingsworth C.L., Randolph A.B., Chong A.Y.L. (2017). An updated and expanded assessment of PLS-SEM in information systems research. Ind. Manag. Data Syst..

[95] Hair J.F., Hult G.T.M., Ringle C.M., Sarstedt M. (2017).

[96] Bagozzi R.P., Yi Y. (1988). On the evaluation of structural equation models. J. Acad. Market. Sci..

[97] Nunnally J.C. (1978).

[98] Hair J.F.J., Hult G.T.M., Ringle C., Sarstedt M. (2014). A primer on partial least squares structural equation modeling. Long. Range Plan..

[99] Henseler J., Ringle C.M., Sarstedt M. (2015). A new criterion for assessing discriminant validity in variance-based structural equation modeling. J. Acad. Market. Sci..

[100] Hair J.F., Ringle C.M., Sarstedt M. (2013). Partial least squares structural equation modeling: rigorous applications, better results and higher acceptance. Lon. Ran. Plan..

[101] Henseler J., Hubona G., Ray P.A. (2016). Using PLS path modeling in new technology research: updated guidelines. Ind. Manag. Data Syst..

[102] Hu L., Bentler P.M. (1999). Cutoff criteria for fit indexes in covariance structure analysis: conventional criteria versus new alternatives. Struct. Equ. Model.: A Mult.J..

[103] Shmueli G., Koppius O.R. (2011). Predictive analytics in information systems research. MIS Q..

[104] Geisser S. (1974). A predictive approach to the random effects model. Biomolecules.

[105] Stone M. (1974). Cross-validatory choice and assessment of statistical predictions. J. Roy. Stat. Soc..

[106] Hair J.F., Risher J.J., Sarstedt M., Ringle C.M. (2019). When to use and how to report the results of PLS-SEM. Eur. Bus. Rev..

[107] Cohen J. (1988).

[108] Hair J.F., Sarstedt M., Matthews L.M., Ringle C.M. (2016). Identifying and treating unobserved heterogeneity with FIMIX-PLS: part I – method. Eur. Bus. Rev..

[109] Islam A. (2015). Investigating e-learning system usage outcomes in the university context. Comp. Educ..

[110] Kim H., Hong A., Song H. (2018). The relationships of family perceived digital competence and attitude and learning agility in sustainable student engagement in higher education. Sustainability.

[111] Martin F., Ahlgrim-Delzell L., Budhrani K. (2017). Systematic review of two decades (1995 to 2014) of research on synchronous online learning. Am. J. Dist. Educ..

[112] Ryan R.M., Deci E.L. (2001). On happiness and human potential: a review of research on hedonic and eudaimonic well-being. Annu. Rev. Psychol..

[113] Wang Y., Chi I., Zhan Y., Chen W., Li T. (2021). Effectiveness of resilience interventions on psychosocial outcomes for persons with neurocognitive disorders: a systematic review and meta-analysis. Front. Psych..

[114] Sparks C., Dimmock J., Lonsdale C., Jackson B. (2016). Modeling indicators and outcomes of students' perceived teacher relatedness support in high school physical education. Psychol. Sport Exerc..

[115] Standage M., Duda J.L., Ntoumanis N. (2005). A test of self‐determination theory in school physical education. Br. J. Educ. Psychol..

[116] Chiu T.K. (2022). Applying the self-determination theory to explain student engagement in online learning during the covid-19 pandemic. J. Res. Technol. Educ..

[117] Kaplan H., Madjar N. (2017). The motivational outcomes of psychological need support among pre-service teachers: multicultural and self-determination theory perspectives. Fron. in Educ..

[118] Martinek D., Carmignola M., Müller F.H., Bieg S., Thomas A., Eckes A., Großmann N., Dittrich A.K., Wilde M. (2021). How can students feel more vital amidst severe restrictions? Psychological needs satisfaction, motivational regulation and vitality of students during the coronavirus pandemic restrictions. Eur. J. Invest. Heal. Psych. Educ..

[119] Jang H., Reeve J., Deci E.L. (2010). Engaging students in learning activities: it is not autonomy support or structure but autonomy support and structure. J. Educ. Psychol..

[120] VicHealth (2015).

[121] Bouchey B., Gratz E., Kurland S. (2021). Remote student support during Covid-19: perspectives of chief online officers in higher education. Online Learn..

[122] Peels R. (2019). Replicability and replication in the humanities. Res. Int. Pee. Rev..

